# NPFs-mediated actin cytoskeleton: a new viewpoint on autophagy regulation

**DOI:** 10.1186/s12964-023-01444-2

**Published:** 2024-02-12

**Authors:** Yuan Dong, Chengshi Quan

**Affiliations:** The Key Laboratory of Pathobiology, Ministry of Education, College of Basic Medical Sciences, Jilin University, 126 Xinmin Avenue, ChangchunJilin, 130021 China

**Keywords:** Autophagy, Actin cytoskeleton, Nucleation-promoting factors, Autophagosome formation, Autophagic lysosome reformation

## Abstract

Macroautophagy/autophagy is a lysosome-dependent catabolic process induced by various cellular stress conditions, maintaining the homeostasis of cells, tissues and organs. Autophagy is a series of membrane-related events involving multiple autophagy-related (ATG) proteins. Most studies to date have focused on various signaling pathways affecting ATG proteins to control autophagy. However, mounting evidence reveals that the actin cytoskeleton acts on autophagy-associated membranes to regulate different events of autophagy. The actin cytoskeleton assists in vesicle formation and provides the mechanical forces for cellular activities that involve membrane deformation. Although the interaction between the actin cytoskeleton and membrane makes the role of actin in autophagy recognized, how the actin cytoskeleton is recruited and assembles on membranes during autophagy needs to be detailed. Nucleation-promoting factors (NPFs) activate the Arp2/3 complex to produce actin cytoskeleton. In this review, we summarize the important roles of the actin cytoskeleton in autophagy regulation and focus on the effect of NPFs on actin cytoskeleton assembly during autophagy, providing new insights into the occurrence and regulatory mechanisms of autophagy.

Video Abstract

**Supplementary Information:**

The online version contains supplementary material available at 10.1186/s12964-023-01444-2.

## Background

Autophagy is a lysosome-dependent catabolic process induced by stress conditions such as starvation, infection and hypoxia, which deliver aggregated proteins, damaged organelles or pathogens to the lysosome for degradation [1, 2]. This self-eating process mediated by the lysosome maintains cellular metabolism and survival during stress conditions on the one hand, and eliminates damaged proteins and organelles on the other hand [3]. Autophagy is therefore important for maintaining the homeostasis of cells, tissues and organs [4]. Dysregulated autophagy has been implicated in a variety of cancer and chronic inflammatory diseases such as inflammatory bowel disease and systemic lupus erythematosus [5]. Thus, revealing the regulatory mechanism of autophagy has broad implications for various human diseases. There are three defined types of autophagy: macro-autophagy, micro-autophagy, and chaperone-mediated autophagy [6]. In micro-autophagy, the lysosomal membrane protrudes or invaginates directly to encapsulate and absorb the cargo [7]. Molecular chaperone-mediated autophagy does not rely on membrane structures to segregate the cargo, but instead uses molecular chaperones to recognize proteins containing specific pentapeptide motifs, and these substrates are subsequently transported directly across the lysosomal membrane [8]. Macro-autophagy segregates cytoplasmic cargoes through a double-membrane structure referred to as autophagosome and degrades the contents by fusion with the lysosome to form the autolysosome. Macroautophagy has been studied most extensively and is hereafter referred to as autophagy.

Autophagy is a complex process involving a variety of autophagy-related (ATG) proteins, which are regulated by a variety of intracellular signaling pathways. For example, mTOR binds to the Ser-757 site of ULK1 and maintains ULK1 in an inactivated state. When stimulated by starvation, ULK1 dissociates from mTOR and initiates autophagy [9]. Decreased level of ATP: AMP ratio leads to AMPK1 activation, which in turn inhibits mTORC1 or directly induces the phosphorylation of ULK1, VPS34 and beclin1 [10]. These signaling pathways usually act on ATG proteins to regulate autophagy. Recent studies have revealed that the actin cytoskeleton is able to act on the membrane during autophagy to regulate a wide range of processes of autophagy [11, 12]. This mechanism provides some new insights for autophagy regulation from actin dynamics. The details of actin regulating autophagy are still unclear, and it remains to be understood how actin is recruited to autophagosomes or autolysosomes to further assemble. Nucleation-promoting factors (NPFs) have been shown to regulate autophagy through the actin cytoskeleton, so NPFs may hold the key to solving this problem. In this review, we focus on the important roles of the actin cytoskeleton in autophagy regulation and analyze the effects of NPFs on the assembly of the actin cytoskeleton during autophagy from the existing research reports.

## The process of autophagy

**Fig. 1:**
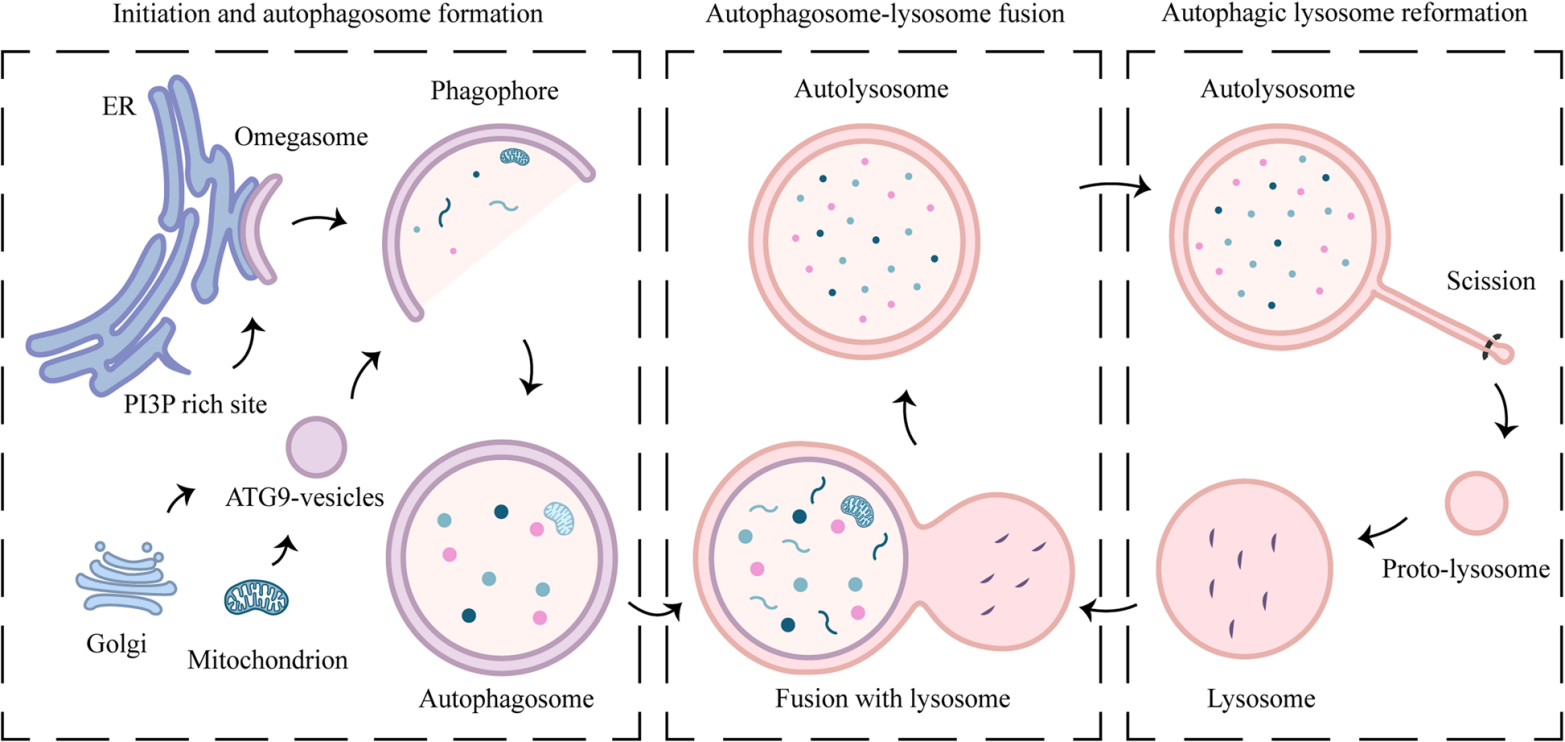
The major steps in autophagy. The events of autophagy include the initiation and autophagosome formation, the fusion of autophagosomes and lysosomes, and ALR. Autophagy starts with the initiation at the omegasome, and the phagophore expands to form the mature autophagosome. ATG9 vesicles transport membrane components of mitochondria and Golgi to facilitate the phagophore expansion. After formation, the autophagosome is translocated and fuses with the lysosome to become the autolysosome. After degradation, autolysosome tubulation generates proto-lysosomes, which finally mature into new lysosomes

Autophagy is a successive, conservative and complex process, including the initiation and autophagosome formation, the fusion of autophagosomes and lysosomes, and autophagic lysosome reformation (ALR) **(**Fig. 1**)** [13, 14]. Several studies have shown that autophagy begins at an endoplasmic reticulum (ER)-associated structure called omegasome in mammals [15, 16]. After initiation, the membrane expands, and the expanded double-membrane sequestering compartment at this stage is called the phagophore [17]. As the phagophore expands, the membrane bends and eventually forms the spherical autophagosome. When the autophagosome is formed, it delivers the cargo to the lysosome. The outer membrane of the autophagosome fuses with the lysosomal membrane to produce the autolysosome, in which the cargo is exposed to lysosomal enzymes for degradation [6]. After degradation, the component parts are exported back into the cytoplasm for use in biosynthetic processes or to generate energy [18]. Lysosomes are extensively consumed during the formation of autolysosomes, and after degradation, lysosomes are recovered from autolysosomes to generate new lysosomes to maintain lysosomal homeostasis in cells [19].

### Initiation and autophagosome formation

The initiation of autophagy can be induced by various intracellular stimuli such as hunger, hypoxia, and oxidative stress, as well as by extracellular stimuli such as mTOR inhibitors [20]. The key to the initiation of autophagy is the activation of the ULK complex, a serine-threonine kinase complex composed of ULK1/2, ATG13, FIP200 and ATG101 [21]. Under normal conditions, activated mTORC1 binds to the ULK complex and blocks the binding of ATG13 to ULK1/2 and FIP200 by phosphorylating ATG13. When stimulated as above, mTORC1 dissociates from the ULK complex, which makes ULK1 autophosphorylated to further activate ATG13 and FIP200 [2, 9, 22]. Next, the ULK complex phosphorylates class III phosphatidylinositol 3-kinase complex I (PI3KC3-C1) (including VPS34, ATG14, beclin1, AMBRA1 and p115), and recruits it to the phagophore assembly site (PAS). ATG13 interacts with ATG14 to anchor the ULK complex to the PAS [1, 2]. VPS34 is the unique member of the PI3KC3, synthesizing phosphatidylinositol 3-phosphate (PI3P) from phosphorylated phosphatidylinositol (PI) [23]. The formation of PI3P is necessary for autophagosome generation in yeast and mammals [24]. ULK1 phosphorylates Ser-15ser15 and 30 sites of beclin1, and the interaction between beclin1 and VPS34 promotes its catalytic activity, leading to an increase in PI3P production [25–27]. Thus, a characteristic PI3P-rich site is formed on the ER called omegasome, which is the "cradle" of autophagosome initiation [28, 29]. The omegasome may be a highly curved region that favors the recruitment of PI3K complex I, which drives further changes in lipid composition and promotes autophagosome formation. This curvature change may also contribute to PI3P exposure and aggregation. Then PI3P recruits its effector molecules WIPI and DFCP1 (also known as ZFYVE1), thereby driving the omegasome to progress towards the phagophore membrane [28, 30–32].

Autophagosome expansion is mediated by two ubiquitin-like coupling systems. The first system involves the formation of the ATG12-ATG5-ATG16L1 complex. Dependent on the effect of the E1-like enzyme, ATG7, and the E2-like enzyme, ATG10, the ubiquitin-like protein ATG12 is conjugatedly coupled to ATG5. Unlike ubiquitination, the coupling of ATG12 to ATG5 in this process is irreversible and does not require the involvement of E3-like ligase [33, 34]. Next, ATG16L1 noncovalently binds to ATG5, and then the ATG12-ATG5-ATG16L1 complex is recruited to the phagophore membrane through the interaction between ATG16L1 and WIPI. After autophagosome formation, the complex dissociates [35–37]. The second ubiquitin-like system is ATG8/LC3. It has been identified multiple ATG8-like proteins in mammals, which are classified into the LC3 and GABARAP subfamilies based on amino acid sequence homology [38]. Among them, the research on LC3 is the most intensive. The cysteine enzyme ATG4 cleaves LC3 into LC3I, exposing its C-terminal glycine residue. In the presence of E1-like enzyme ATG7 and E2-like enzyme ATG3, LC3I is activated and transferred to ATG3. Through the interaction between ATG3 and ATG12, the E3-like enzyme ATG12-ATG5-ATG16L1 complex anchors LC3 to the phagophore membrane. Then the glycine at the end of LC3I covalently binds to phosphatidylethanolamine, which transforms LC3I into the lipidated form, LC3II, thereby promoting phagophore expansion [39–41]. LC3II plays an important role in autophagosome generation by promoting autophagosome membrane extension [42], and its conversion correlates with autophagosome formation. LC3II is considered to be a reliable autophagosome marker [39].

The source of membrane components during autophagy has been a complex issue. In addition to the ER, it has been found that the plasma membrane, mitochondria, endosomes, and the Golgi complex are able to provide membrane components to facilitate the elongation of autophagosome membrane. This process is usually accomplished through ATG9-containing vesicles, which circulate between the phagophore membrane and different cytoplasmic membranes to participate in transportation during autophagosome generation [1, 43].

As the phagophore membrane extends and engulfs its contents, the autophagosome membrane eventually closes. The mechanisms involved in the closure process are still unclear and may depend on the transport of endosomal sorting complex required for transport (ESCRT) mechanism [44, 45]. ESCRT-I is found to be required for autophagosome closure. When recruited to autophagosomes, ESCRT-I assembles into a cyclic complex, recruiting downstream ESCRTs to promote autophagosome closure [46]. Besides, defects in the ATG8 conjugation system can also lead to impaired closure of the autophagosome membrane [47], which suggests that the ATG8 system plays a role in this process, but the specific details require further research.

### Autophagosome-lysosome fusion

After formation, autophagosomes are translocated towards lysosomes and eventually accumulate in the perinuclear region where lysosomes are located [48]. The fusion of autophagosomes and lysosomes is mainly mediated by the SNARE complex. STX17, SNAP29 located on autophagosomes, and VAMP7/VAMP8 located on lysosomes are key proteins involved in this process. In addition, the fusion process also requires the synergistic effect of other proteins such as small GTP enzymes [49, 50]. For example, EPG5, the effector molecule of the RAB GTP enzyme, can bind to LC3 to stabilize the STX17-SNAP29-VAMP7/8 complex, thereby promoting the fusion of autophagosomes and lysosomes [51]. Some ATG proteins have also been shown to play a role in the fusion process, such as ATG14 directly binding to the STX17-SNAP29 complex to promote its fusion with VAMP8 on lysosomes [52]. The fusion of autophagosomes and lysosomes is a complex and delicate process, and its molecular mechanism still needs to be further investigated.

### Autophagic lysosome reformation

After autophagosome-lysosome fusion, autolysosomes are formed. Cargoes carried by autophagosomes are degraded by lysosomal hydrolases, and the degradation products are expelled from autophagosomes through lysosomal transport proteins. During the peak period of autophagy, the level of free lysosomes in cells is consumed by the formation of autolysosomes. Therefore, after autophagy is completed, it is necessary to restore the level of free lysosomes in cells through the ALR [14, 53]. An early and decisive event in the ALR process is autolysosome tubulation, during which a tubular structure composed of lysosomal membrane components is extruded from the autolysosome. The initiation of autolysosome tubulation is to form a bud enriched with phosphatidylinositol 4,5-bisphosphate (PI(4,5)P_2_) and enclosed by clathrin on autolysosome. KIF5B, a member of the kinesin family, is recruited to autophagolysosomes by directly binding to PI(4,5)P_2_, generating a driving force to facilitate the protrusion of tubulation; besides, clathrin promotes the enrichment of PI(4,5)P_2_ and the recruitment of KIF5B, which in turn drives the process of autolysosome tubulation [54, 55]. After tubulation, proto-lysosomes are generated from the top of the tubules and subsequently are cut into new lysosomes under the mediation of PIP5K1A and DNM2. However, the mechanism by which these newly formed lysosomes obtain acidity and mature into new lysosomes remains to be elucidated [14, 56].

In addition to PI(4,5)P_2_, other phospholipids, including PI(4)P and PI(3)P, also participate in different stages of ALR. However, it is still unclear whether different phospholipids synergize or regulate ALR in different ways. Further research is needed to detail the effect of various phospholipids and their upstream kinases in the ALR process [14].

## Actin cytoskeleton and autophagy

Autophagy is a complex series of membrane-related events controlled by multiple proteins, and recent studies have gradually revealed the important regulatory effect of the actin cytoskeleton on various stages of autophagy.

### The structure and function of actin cytoskeleton

The actin cytoskeleton is a dynamic system composed of hundreds of proteins and contributes to maintaining cell shape and polarity and as well as provide power and directionality for a myriad of motile functions in cells. The core component of this system is actin, and this system is mainly formed by different combinations of actin filaments (F-actin) [57]. Globular actin monomers (G-actin) are the basic units to form F-actin. G-actin rapidly elongates on the basis of dimers or trimers, forming a double helix structure of F-actin. The aggregation of actin is a dynamic process. Due to the different extension rates at two ends of F-actin, F-actin has polarity. The more dynamic end is the barbed (or +) end, and elongates 10 times faster than the pointed (or -) end [58, 59]. The polymerization of actin filaments and their combination with actin-binding proteins produces a variety of architectures.

The branched actin network is a specific form of the actin cytoskeleton and is a branched network structure mediated by the Arp2/3 complex, which is the only molecular machine that generates branched actin networks. The Arp2/3 complex is a stable multiprotein complex composed of 7 subunits, with a total mass of ~ 250 kDa. Two of the subunits, Arp2 and Arp3, are actin-related proteins. In the basal state, Arp2 and Arp3 are separated by the other five subunits (ARPC1-5), which maintain the Arp2/3 complex in an inactive state. When activated, Arp2 and Arp3 are brought together so that the conformation of the complex is altered, and this active conformation allows the Arp2/3 complex to bind to F-actin [60]. Arp2 and Arp3 subunits stimulate actin dimers and ARPC1-5 serves as a scaffold subunit to hold them together. The activated Arp2/3 complex binds to the side of the pre-existing actin filaments (actin mother filaments), mediating the nucleation of branched networks and prolonging new actin filaments (daughter filaments) at a specific ~ 70° angle [57, 61].

Branched actin networks assemble at the leading edge of motile cells, driving the protrusion of lamellipodia [62], which is closely related to cell movement [63]. In addition, Arp2/3-mediated branched actin networks generate dynamics that drive various intracellular movements. The earliest research linking the Arp2/3 complex with intracellular movement focused on bacterial pathogens such as Listeria, Shigella, and Rickettsia. On the surface of these pathogens, F-actin and actin-related proteins in host cells form the "comet tail", which is mainly composed of Arp2/3-mediated branched actin networks. Driven by this structure, pathogens move within and between cells [64, 65]. The comet tail structure also drives the transport of endosomes in cells, and branched actin networks can also deform or invaginate the plasma membrane, providing membrane curvature and participating in multiple stages of endocytosis [64, 66]. Although different studies have been conducted on the phenomenon that branched actin networks are capable of generating dynamical properties, the specific details of actin networks driving the movement of the target object still need to be further investigated in depth [58].

### The regulation of autophagy by actin cytoskeleton

The ability of actin to polymerize and branch provides mechanical forces in a variety of cellular activities associated with membrane deformation, such as endocytosis and phagocytosis [67, 68]. In view of the function of the branched actin network in membrane-related events, in recent years more and more studies have gradually revealed the importance of the branched actin network in the autophagy process.

As early as 1992, it was observed that starved rat kidney cells treated with actin depolymerizing agents failed to produce autophagosomes [69]. Subsequent studies in yeast cells found that the effect of actin on non-selective autophagy is optional, but in selective autophagy, actin is necessary for cargo selection [70–72]. In mammalian cells, actin was initially found to be involved in the early stages of autophagy. Treatment with actin-depolymerizing drugs such as Latrunculin B inhibits autophagosome production under starvation [73], and treatment with Cytochalasin D interferes with autophagosome maturation [74]. Besides the effects of actin-depolymerizing drugs on autophagy, colocalization of F-actin with several important ATG proteins has been observed. During starvation-induced autophagy, F-actin colocalizes with ATG14, DFCP1 and beclin1, key proteins in the early events of autophagosome formation [73], and with LC3 [75]. As the puncta showing positive fluorescence staining for both the autophagosome marker LC3 and the lysosome marker LAMP1 are considered to be phagophore, while LC3-positive and DFCP1-negative puncta are considered to be mature autophagosomes. It was observed that F-actin colocalizes with ATG proteins such as ATG5, ULK1, ATG14 and ATG16 in phagophore [75].

Although these studies have demonstrated the participation of actin in autophagy, how actin is involved in this process remains unclear. Until recently, actin branching and forming networks to regulate autophagy has been gradually revealed. Similar to the role of branched actin networks on vesicles in endocytosis, during starvation-induced autophagy, actin networks shape autophagosomes from inside and outside the phagophore. During autophagy initiation, actin is not detected at the ER sites specific to autophagosome formation. After omegasome formation, actin is recruited to assemble inside and outside the autophagosome, stabilizing and promoting membrane curvature. It is hypothesized that the effect is due to actin networks acting as the scaffold or generating propulsion force to maintain the high curvature required for phagophore expansion [11, 75]. Apart from its impact on the formation of autophagosomes, mature autophagosomes rely on the comet tail mechanism formed by branched actin networks for transport in the cytoplasm, and the actin cytoskeleton is necessary for the fusion of autophagosomes and lysosomes. ATG9 vesicles transport membrane components to autophagosomes during autophagy, and the assembly of the actin cytoskeleton on ATG9A vesicles is critical for the proper trafficking of ATG9A vesicles from endosomes to autophagosomes [12, 76].

The formation of branched actin networks relies on the mediation of the Arp2/3 complex. Indeed in studies of the involvement of the actin cytoskeleton in autophagy, the importance of Arp2/3 in autophagy has been identified. Cells treated with CK666, a cell permeability drug that inhibits Arp2/3 activation [77], lead to decreased LC3II levels and reduced volume and quantity of autophagosomes [78, 79]. Inhibition of Arp2/3 activation interferes with the bending of the phagophore, leading to the collapse of the omegasome [75]. During phagophore expansion, the cycle of ATG9-containing vesicles is dependent on the Arp2/3 complex, and mutations in Arp2 block selective and non-selective autophagy in yeast cells [80]. After CK666 treatment, the colocalization of ATG9 and F-actin, as well as ATG9 and LC3, was decreased, indicating that the transport of ATG9 vesicles by actin networks during autophagy depends on the mediation of the Arp2/3 complex [81]. Autolysosome tubulation, the vital event in ALR, is inhibited after CK666 treatment and restored after CK666 elution [82].

Although a large amount of research has shown that branched actin networks play an important role in multiple different stages of autophagy, how actin is recruited to autophagosomes or autolysosomes and then assembles into branched networks remains a question that needs to be studied. Given the role of Arp2/3 in the regulation of autophagy by actin networks, it has been discovered that NPFs responsible for Arp2/3 complex activation participate in autophagy. NPFs may become the crux of controlling actin networks during autophagy.

## NPFs and autophagy

### The regulation of branched actin networks by NPFs

**Fig. 2:**
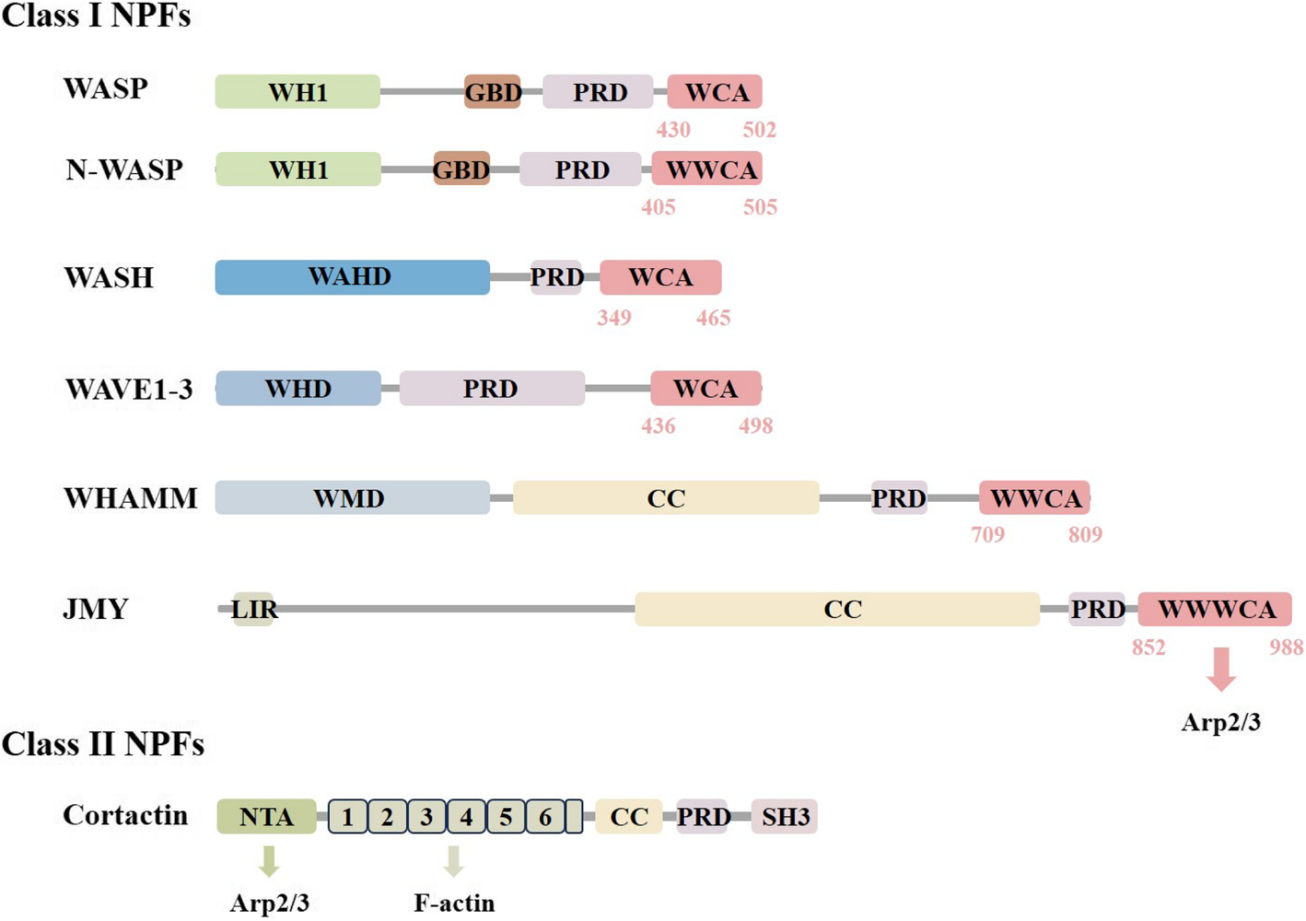
Structural organization of NPFs. Class I NPFs contain WASP, WASH, WAVE, WHAMM, and JMY, which have characteristic WCA domains at the C-terminal. WCA is the smallest unit of the efficient activation of the Arp2/3 complex, with the WH2 domain capable of binding to G-actin and the CA domain responsible for binding to Arp2/3. The main member of class II NPFs is cortactin, without WCA domains. NTA domain binds to the Arp2/3 complex, and the tandem repeat domain binds to F-actin. The N-terminal domains of NPFs are different and can provide binding sites for a wide range of regulatory factors in cells. WH1, WASP homology1; WAHD, WASH homology domain; WHD, WAVE homology domain; WMD, WHAMM membrane interaction domain; LIR, LC3-interacting region; GBD, GTPase binding domain; PRD, proline-rich-domain; CC, coiled-coil; W, WASP homology2 (WH2) domain; C, central domain; A, acidic domain; NTA, N-terminal acidic domain; SH3, Src homology 3 domain

NPFs are activators of the Arp2/3 complex and can be divided into two main types according to their structure **(**Fig. 2**)**. Class I NPFs contain the following four families: Wiskott-Aldrich syndrome protein (WASP), WASP homolog (WASH), WASP-family verprolin homolog (WAVE) and WASP homolog associated with actin, membranes and microtubules (WHAMM). The WASP family is composed of two paralogous proteins, WASP and neural WASP (N-WASP). The WAVE family consists of three WAVE proteins (WAVE1-3). The WHAMM family contains its homologous protein, junction-mediating regulatory protein (JMY). These proteins have characteristic WCA domains at the C-terminal, including the WH2 domain (WASP-homology 2 domain, W), the central domain (C), and the acidic domain (A), WCA is the smallest unit of the efficient activation of the Arp2/3 complex, with the WH2 domain capable of binding to G-actin and the CA domain responsible for binding to Arp2/3 [83–85]. Generally, the N-terminal domains of NPFs are different and can provide binding sites for a wide range of regulatory factors in cells [86]. Class II NPFs do not contain WCA domains, and their main members are cortactin. Cortactin contains the following domains: N-terminal acidic domain (NTA), tandem repeat domain, proline-enriched domain, and C-terminal Src homology 3 domain (SH3 domain). Among them, the proline-enriched region contains multiple phosphorylation sites, and the SH3 domain can bind to various cytoskeletons or signaling proteins. The N-terminal of cortactin is responsible for regulating the assembly of branched actin networks. NTA domain binds to the Arp2/3 complex, and the tandem repeat domain binds to F-actin [83, 87].

**Fig. 3:**
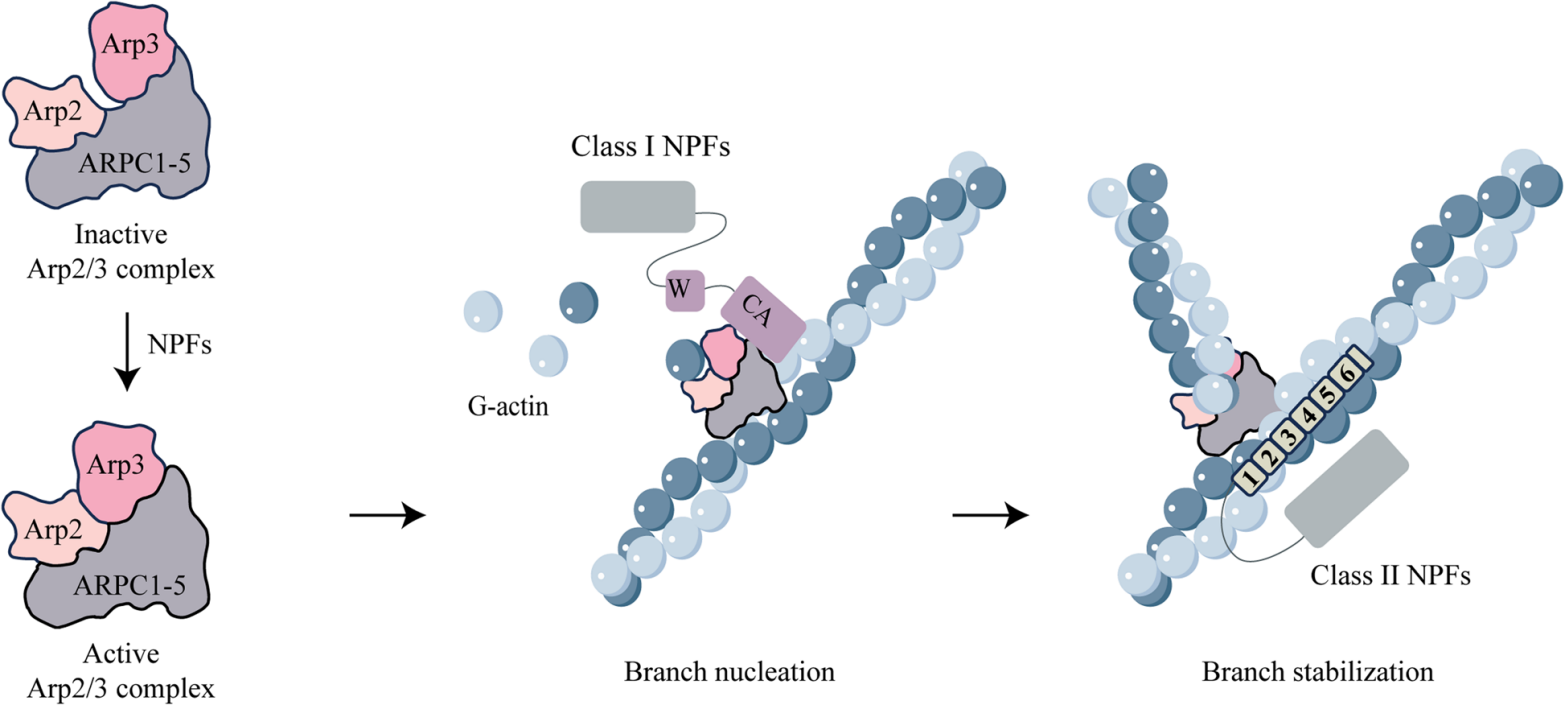
The model for the activation of the Arp2/3 complex mediated by NPFs and the formation of the branched actin network mediated by NPFs. NPFs lead to conformational change and activation of the Arp2/3 complex. Class I NPFs bring the activated Arp2/3 complex together with the actin mother filaments via WCA, mediating the nucleation of daughter filaments. Class II NPFs stabilize Arp2/3 branchpoints and alter the conformation of F-actin to increase the affinity of the Arp2/3 complex for actin mother filaments

Class I NPFs rely on the WCA domain to activate the Arp2/3 complex **(**Fig. 3**)**. The CA region acts on multiple subunits of the complex, leading to conformational change and activation of the Arp2/3 complex. Current research on its structure has shown that the central domain can bind to the Arp2 subunit. The activated Arp2/3 complex binds to the side of actin mother filaments, mediating the nucleation of daughter filaments [57, 88, 89]. The WH2 domain binds G-actin monomers and delivers them to the nucleation site to generate new daughter filaments. After the Arp2/3 complex is activated, class I NPFs dissociate and mediate a new process of nucleation [88].

Class II NPFs activate Arp2/3 weakly, but regulate branched actin network assembly can be regulated through various mechanisms **(**Fig. 3**)**. Cortactin can bind to WASP family proteins to enhance the activation of Arp2/3 by WASP [90, 91]. Structural studies have shown that cortactin can alter the conformation of F-actin, thereby increasing the affinity of the Arp2/3 complex for actin mother filaments [87, 92]. In addition, the unique and important function of cortactin is to stabilize the Arp2/3 mediated branch and prevent branched network dissociation [93].

### The regulation of autophagy by NPFs

Given the indispensable role of NPFs in Arp2/3 complex activation and branched actin network formation, recent studies have gradually revealed the important role of NPFs in autophagy. Different members of the NPFs family involved in different events of autophagy are summarized in Table 1 and discussed in further detail below.

**Table 1:** The function and mechanism of NPFs in autophagy

NPFs	Effects on autophagy	Underlying mechanisms	Stimulating factors	References
WASP	Promotes autophagosome generation	Via actin cytoskeleton	*E. coli* infection Rapamycin treatment	[94] [95]
	Promotes autophagosome-lysosome fusion	Via actin cytoskeleton	Rapamycin treatment	[95]
WASH	Promotes the transport of ATG9 vesicles	Unknown	Starvation	[96]
	Negatively regulates autophagy	Acts on beclin1 and PI3KC3	Starvation	[97]
WAVE	Promotes autophagosome generation	Unknown	Adriamycin treatment	[98]
WHAMM	Promotes autophagosome generation	Via actin cytoskeleton	Starvation	[79]
	Promotes autophagic lysosome reformation	Binds to PI(4,5)P_2_ to promote actin cytoskeleton assembly	Starvation	[82]
JMY	Promotes autophagosome generation	Binds to LC3 to promote actin cytoskeleton assembly	SAHA treatment or starvation Starvation	[78] [99]
	Participates in late phases of autophagy	Unknown	Starvation	[99]
Cortactin	Promotes autophagosome-lysosome fusion	Via actin cytoskeleton	MG132 treatment None	[100] [101]

#### WASP

WASP is the original member of the class I NPF family, a protein mutated in a rare immunodeficiency called Wiskott–Aldrich syndrome (WAS) [102]. WASP is specifically expressed in the immune system, and its homologous N-WASP is widely expressed in various tissues [103]. WASP/N-WASP activates Arp2/3 through the C-terminal WCA domain and mediates actin polymerization. In resting cells, WASP and N-WASP are in a closed inactive auto-inhibited conformation. WASP-interacting protein (WIP) contributes to regulating the activation and stability of WASP, participates in the reorganization of the actin cytoskeleton, and stabilizes actin filaments in a WASP-dependent or independent manner [104]. Lee et al. found that WASP regulated autophagy in dendritic cells infected with *E. coli*, in which WASP knockout results in decreased expression of LC3II, and the disappearance of colocalization of F-actin with LC3 and p62. These data indicate that WASP participates in autophagy through actin [94]. Recent research by Rivers et al. further confirms the role of WASP in autophagy. WASP knockout inhibits the generation of autophagosomes in the monocytic cell line, THP-1, and damaged autophagosome generation is also observed in human monocyte-derived macrophages (MDMs) extracted from WAS patients compared to healthy donors. The absence of WASP disrupts the colocalization of LC3 and LAMP1 and makes them dispersedly distributed. The colocalization of LC3 and F-actin is also reduced without WASP. After gene therapy to undergo in vitro WASP reconstitution, LC3 and LAMP1 are colocalized in the perinuclear region, and the colocalization of LC3 and F-actin is restored in MDMs. These data indicate that WASP relies on actin to participate in the fusion of autophagosomes and lysosomes [95]. CK666 treatment or ARPC1B (one of the subunits of Arp2/3 complex) knockdown causes functional defects in Arp2/3, reducing the transformation of LC3II and the formation of LC3 puncta, and interfering with the colocalization of LC3 puncta and F-actin, which further prove that WASP regulates autophagy via Arp2/3-mediated actin cytoskeleton [95].

In our previous study, we found that the WIP-dependent actin cytoskeleton is critical for CLDN6-induced autophagy. WIP colocalizes with LC3 and p-Arp3; breast cancer cells treated with CK666 exhibit reduced autophagic flux. Besides, WIP knockdown results in reduced LC3II expression and the number of autophagosomes, and makes the actin polymerizer Jaslakinolide lose its regulatory effect on autophagy, which indicates that actin cytoskeleton dependent on WIP to regulate autophagy [105]. WIP is not a member of NPFs in the traditional definition, but it affects actin assembly by regulating multiple NPFs (WASP, N-WASP, and cortactin) [104].

#### WASH

WASH is an important regulatory factor for vesicular transport in cells, directly regulating the sorting and maturation of endosomes by generating actin networks on the surface of vesicles [106, 107]. In Dictyostelium, WASH does not affect autophagosome formation but is involved in lysosomal digestion and degradation of autophagic cargoes as well as lysosomal enzyme recycling [108]. In mammals, WASH is necessary for the transport of ATG9 during autophagy. WASH knockdown decreases the colocalization of LC3 and ATG9, the transformation of LC3II, and the number of LC3 puncta, which indicates that WASH promotes autophagy and ATG9 transport [96]. The transport of ATG9 vesicles depends on the actin comet tail [12], which derives a speculation that WASH may regulate autophagy via the actin network. However, the study did not elucidate this speculation, and further data are required to support it [96]. Some studies have also reported that WASH negatively regulates autophagy by acting on autophagy-associated proteins. Xia et al. demonstrated that ubiquitination of beclin1 is required for autophagy induction and that WASH competitively binds to beclin1 to inhibit its ubiquitination, thereby negatively regulating autophagy [97]. In addition, WASH promotes the ubiquitination degradation of AMBRA1, the key protein of PI3KC3, to inhibit autophagy [109]. These data suggest that there may be diversified effects of WASH on the autophagic process, which may not just depend on the actin cytoskeleton.

#### WAVE

WAVE consists of three isoforms: WAVE1, WAVE2, and WAVE3. The activation of Arp2/3 by the WAVE family promotes actin polymerize, which is crucial for membrane protrusion events such as the formation of pseudopodia [110]. Research on WAVE and autophagy was limited. Zhang et al. found that silencing WAVE1 decreases the ratio of LC3II to LC3I and the number of autophagosomes under electron microscopy, indicating that WAVE1 promotes the generation of autophagosomes [98]. However, the specific mechanism is still unclear.

#### WHAMM

WHAMM contains two WH2 domains that can stimulate Arp2/3 mediated actin assembly. In addition to its effects on Arp2/3 and actin, WHAMM can also bind to microtubules. WHAMM has been shown to play a role in ER-Golgi transport in cells [111]. In 2015, Kast et al. reported the role of WHAMM in autophagosome generation. They found that WHAMM colocalizes and comigrates with autophagy markers DFCP1, LC3, and p62, and Arp2/3 mediated actin comet tail drives the movement of these puncta. Inhibition of Arp2/3 mediated comet tails assembly reduces the number and volume of autophagosomes. These data indicate that WHAMM promotes the generation of autophagosomes through the Arp2/3-mediated branched actin network [79]. The authors did not find colocalization between WHAMM and ATG14 prior to omegasome formation, as well as with LAMP1 after autophagosomes fuse with lysosomes. Therefore, the authors suggest that WHAMM is involved in the early events of autophagy after the formation of omegasome [79]. However, in subsequent studies, Dai et al. confirmed that instead of influencing autophagosome biogenesis, WHAMM is mostly involved in ALR. This study found that WHAMM colocalizes with the autolysosome that is positive for both the autophagosome marker LC3 and the lysosome marker LAMP1, and promotes autolysosome tubulation by generating branched actin networks on autolysosomes. WHAMM knockout does not affect the number and size of autophagosomes [82]. The main reason for the differences between these two research reports may be the different cell lines chosen for the study, which suggests that the WHAMM participates in different stages of autophagy in different cells and regulates autophagy via branched actin networks.

#### JMY

JMY was initially identified as a cofactor for the transcriptional regulator p300/CBP, regulating p53 activity [112], and subsequent studies have confirmed that JMY is a multifunctional NPF [113]. JMY contains three WH2 domains, which can promote actin nucleation through Arp2/3-dependent or independent methods [102]. JMY is localized to dynamic vesiculo-tubular structures that are decorated with actin and Arp2/3 complex throughout the cytoplasm, and drives actin-dependent vesicle transport away from the trans-Golgi surface [114]. Coutts et al. revealed that JMY is located on autophagosomes and promotes actin nucleation to regulate the formation of autophagosomes. The study observed colocalization of LC3 and F-actin on autophagosomes, and treatment with Arp2/3 inhibitors decreases the ratio of LC3II to LC3I and the number of autophagosomes [78]. Recent studies have elucidated the role of JMY in autophagy in further detail. JMY is recruited to autophagosomes after the formation of PI3P-rich sites and JMY colocalizes with DFCP1, ATG9, and LAMP1. Under fluorescence microscopy, the puncta that are positive for both LC3 and JMY are moved in the cytoplasm driven by the Arp2/3-mediated actin comet tail, and the similar phenomenon is further observed when this process is reconstructed in vitro using purified proteins. These data indicate that JMY is involved in multiple events during the formation of autophagosomes. Moreover, the fact that JMY colocalizes with LAMP1 suggests that JMY activity might play a unique role in some late phases of autophagy [99].

#### Cortactin

Cortactin assumes a variety of functions in various cell types, all of which are largely dependent on its effects on the Arp2/3 complex and actin cytoskeleton [87]. Lee et al. found that cortactin knockdown inhibits autophagosome-lysosome fusion and that this effect of cortactin is dependent on the assembly of actin networks [100]. Subsequent studies further demonstrated that activated cortactin stabilizes F-actin on lysosomes, which is necessary for the fusion of autophagosomes and lysosomes [101]. Cortactin is regulated by post-translational modifications, and the phosphorylation of certain specific sites is related to its cellular function. For instance, the phosphorylation of cortactin by Src at Y421 and Y470 sites is associated with cell migration and invasion, and the acetylation of cortactin is negatively correlated with its actin-binding ability [115]. It was found that phosphorylated cortactin enhances its activity and promotes autophagosome-lysosome fusion [101], while acetylated cortactin cannot support the fusion of autophagosomes and lysosomes [100].

**Fig. 4:**
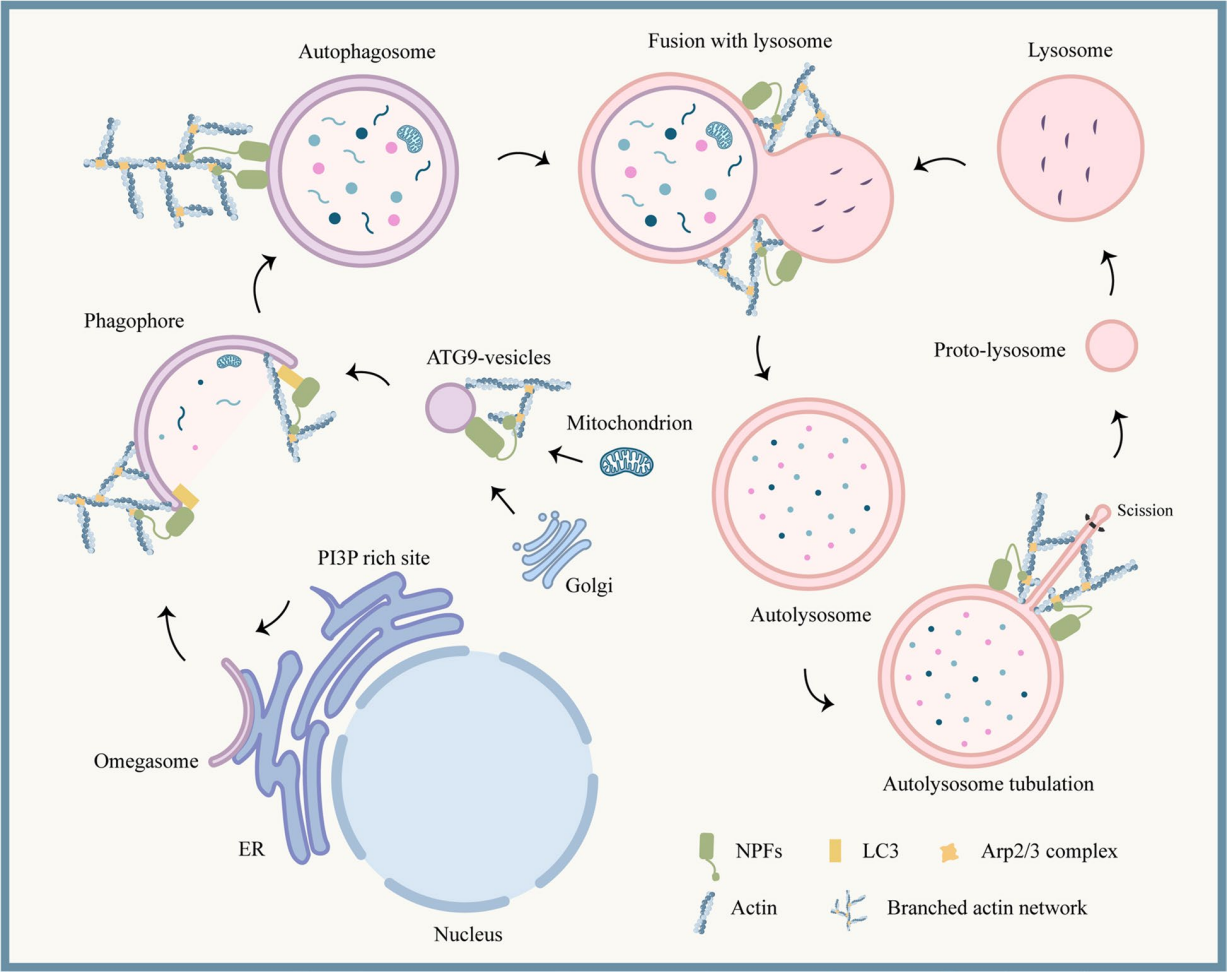
The involvement of NPFs and actin cytoskeleton in different events of autophagy. NPFs mainly depend on the assembly of Arp2/3-mediated actin cytoskeleton to participate in the generation of autophagosomes after the formation of omegasome, the transport of ATG9 vesicles and autophagosomes, the fusion of autophagosomes and lysosomes, and the ALR process. LC3 and phosphoinositides on autophagosome or autolysosome membranes may be the sites where NPFs anchor, and this binding may be a necessary process for NPFs to participate in autophagy

The above studies elucidate the important role of NPFs dependent on the actin network in multiple events of autophagy, and provide a basis for NPFs to become key factors mediating actin cytoskeleton assembly on autophagosomes and autolysosomes. However, existing studies have not fully elucidated how NPFs are anchored to autophagosomes. JMY contains the LC3-interacting region (LIR) motif [99], and proteins containing this motif can directly interact with LC3 protein [116]. The proteins reconstructed in vitro further prove that the LC3 protein combined with liposomes (simulating LC3 on autophagosome membranes) can bind to JMY, which enhances the ability of JMY to activate the Arp2/3 complex and promote actin assembly [99]. Additionally, in the process of promoting ALR, WHAMM is recruited by PI(4,5)P_2_ on autolysosomes through two amphiphilic helical structures at the N-terminal. When these structures mutate, WHAMM is unable to locate on autolysosomes, and the ALR process is blocked [82]. These limited studies suggest that LC3 and specific phosphoinositides on autophagosome or autolysosome membranes may be the sites where NPFs anchor, and this binding may be a necessary process for NPFs to participate in autophagy. Although these speculations still require further extensive research, existing studies have revealed the significance of NPFs in the assembly of branched actin networks during autophagy **(**Fig. 4**)**.

### The regulation of NPFs and autophagy by stimulating factors

In studies of NPFs regulating autophagy, some stimuli are often used to induce autophagy, which has been summarized in Table 1. Dai et al. found that after 2 h of starvation, WHAMM knockout can not affect the generation of autophagosomes. However, after 8 or 12 h of starvation, it was observed that WHAMM knockout affects ALR and causes accumulation of enlarged autolysosomes [82]. This is because different starvation time leads to different events of autophagy, and WHAMM plays a role in the late stages of autophagy. Besides, Lee et al. found that cortactin and actin have little effect if autophagosomes and lysosomes were purified from starved cells, implying that cortactin-mediated F-actin is not involved in nutrient-independent autophagy [100]. These results suggest that different stimuli and treatment time may need to be considered in the regulation of autophagy by NPFs. However, Hasegawa et al. confirmed that cortactin-mediated F-actin regulates autophagy without considering the influence of stimuli [101]. In our previous research, we found that WIP-mediated F-actin regulates autophagy without stimuli [105]. These studies indicated that NPFs can also regulate autophagy independently of stimuli.

Some studies have found that under hypoxic stimulation, HIF-1α regulates the expression of JMY and N-WASP through the hypoxia response element in their promoter [117, 118]. During hypoxia, HIF-1α regulates multiple ATG proteins to induce autophagy. In addition to regulating the expression of beclin1 at the transcriptional level [119], HIF-1α promotes translation of ATG2A and ATG14 in a m6A-dependent manner [120]. Therefore, in future research, the regulation of stimuli on NPFs and autophagy at the transcriptional and post-transcriptional levels should be considered.

## Conclusions and future perspectives

Autophagy is a complex and precise process involving a variety of ATG proteins. It plays a critical role in maintaining homeostasis and regulating human diseases. Thus, uncovering the regulatory mechanism of autophagy is essential for various diseases. Most studies on autophagy regulation have focused on ATG proteins affected by signaling pathways. Recently, the role of the actin cytoskeleton in autophagy-associated membranes illustrates the regulatory mechanism of autophagy from a new perspective. Nonetheless, there are still some unresolved questions. It is unknown how actin cytoskeleton is recruited and assembles on membranes during autophagy.

In this review, we summarized the regulatory role of NPFs in multiple events of autophagy. The same NPFs may participate in different autophagy processes in different cell lines. NPFs participate in the generation of autophagosomes after the formation of omegasome, the transport of ATG9 vesicles and autophagosomes, the fusion of autophagosomes and lysosomes, and the ALR process. Most relevant studies have shown that the regulation of autophagy by NPFs depends on the assembly of the actin cytoskeleton. However, some studies have found that NPFs negatively regulate autophagy by acting on ATG proteins, indicating that the role of NPFs in autophagy may be diverse and not just dependent on the actin cytoskeleton. LC3 or specific phosphoinositides on autophagosomes or autolysosomes can recruit NPFs to the membrane to promote the assembly of branched actin networks. These studies elucidate the important regulatory role of NPFs and actin cytoskeleton in autophagy, and provide a basis for NPFs as the core of actin cytoskeleton assembly in mediating autophagy. In the future, detailed studies on the NPFs-mediated actin cytoskeleton will offer new insights into the occurrence and regulatory mechanism of autophagy, which will enable a better understanding of this important and complex process. Since autophagy is a major degradation process in cells, recent studies have also observed the degradation of actin-regulatory proteins targeted by autophagy. The accumulation of scinderin and kindlin3 caused by autophagy damage leads to disorganization or disassembly of F-actin [121, 122]. These studies suggest that the relationship between actin cytoskeleton and autophagy may not be simply a unilateral regulation. Therefore, future research should take into account possible interactive regulation between actin cytoskeleton and autophagy.

## Data Availability

Not applicable.

## Abbreviations

ATG: Autophagy-related
NPFs: Nucleation-promoting factors
ALR: Autophagic lysosome reformation
ER: Endoplasmic reticulum
PI3KC3-C1: Class III phosphatidylinositol 3-kinase complex I
PAS: Phagophore assembly site
PI3P: Phosphatidylinositol 3-phosphate
PI: Phosphatidylinositol
ESCRT: Endosomal sorting complex required for transport
PI(4,5)P_2_: Phosphatidylinositol 4,5-bisphosphate
F-actin: Actin filaments
G-actin: Globular actin
WASP: Wiskott-Aldrich syndrome protein
WASH: WASP homolog
WAVE: WASP-family verprolin homolog
WHAMM: WASP homolog associated with actin, membranes and microtubules
JMY: Junction-mediating regulatory protein
WH2: WASP-homology 2
NTA: N-terminal acidic domain
SH3: Src homology 3
WAS: Wiskott–Aldrich syndrome
N-WASP: Neural WASP
WIP: WASP-interacting protein
MDMs: Monocyte-derived macrophages
LIR: LC3-interacting region

## References

[1] Dikic I, Elazar Z (2018). Mechanism and medical implications of mammalian autophagy. Nat Rev Mol Cell Biol.

[2] Li X, He S, Ma B (2020). Autophagy and autophagy-related proteins in cancer. Mol Cancer.

[3] Rabinowitz JD, White E (2010). Autophagy and metabolism. Science (New York, NY).

[4] Levine B, Kroemer G (2019). Biological functions of autophagy genes: a disease perspective. Cell.

[5] Don Wai Luu L, Kaakoush NO, Castaño-Rodríguez N. The role of ATG16L2 in autophagy and disease. Autophagy. 2022;18(11):2537–46. 10.1080/15548627.2022.2042783 PMC9629082 35239457

[6] Parzych KR, Klionsky DJ (2014). An overview of autophagy: morphology, mechanism, and regulation. Antioxid Redox Signal.

[7] Mijaljica D, Prescott M, Devenish RJ (2011). Microautophagy in mammalian cells: revisiting a 40-year-old conundrum. Autophagy.

[8] Massey A, Kiffin R, Cuervo AM (2004). Pathophysiology of chaperone-mediated autophagy. Int J Biochem Cell Biol.

[9] Hosokawa N, Hara T, Kaizuka T, Kishi C, Takamura A, Miura Y (2009). Nutrient-dependent mTORC1 association with the ULK1-Atg13-FIP200 complex required for autophagy. Mol Biol Cell.

[10] Kim J, Kundu M, Viollet B, Guan KL (2011). AMPK and mTOR regulate autophagy through direct phosphorylation of Ulk1. Nat Cell Biol.

[11] Zientara-Rytter K, Subramani S (2016). Role of actin in shaping autophagosomes. Autophagy.

[12] Kruppa AJ, Kendrick-Jones J, Buss F (2016). Myosins, actin and autophagy. Traffic (Copenhagen, Denmark).

[13] Li YJ, Lei YH, Yao N, Wang CR, Hu N, Ye WC (2017). Autophagy and multidrug resistance in cancer. Chin J Cancer.

[14] Chen Y, Yu L (2017). Recent progress in autophagic lysosome reformation. Traffic (Copenhagen, Denmark).

[15] Hayashi-Nishino M, Fujita N, Noda T, Yamaguchi A, Yoshimori T, Yamamoto A (2009). A subdomain of the endoplasmic reticulum forms a cradle for autophagosome formation. Nat Cell Biol.

[16] Ylä-Anttila P, Vihinen H, Jokitalo E, Eskelinen EL (2009). 3D tomography reveals connections between the phagophore and endoplasmic reticulum. Autophagy.

[17] He C, Klionsky DJ (2009). Regulation mechanisms and signaling pathways of autophagy. Annu Rev Genet.

[18] Yorimitsu T, Klionsky DJ. Autophagy: molecular machinery for self-eating. Cell death and differentiation. 2005;12 Suppl 2(Suppl 2):1542–52. 10.1038/sj.cdd.4401765 PMC1828868 16247502

[19] Nanayakkara R, Gurung R, Rodgers SJ, Eramo MJ, Ramm G, Mitchell CA (2023). Autophagic lysosome reformation in health and disease. Autophagy.

[20] Mizushima N (2007). Autophagy: process and function. Genes Dev.

[21] Xiang H, Zhang J, Lin C, Zhang L, Liu B, Ouyang L (2020). Targeting autophagy-related protein kinases for potential therapeutic purpose. Acta Pharmaceutica Sinica B.

[22] Jung CH, Jun CB, Ro SH, Kim YM, Otto NM, Cao J (2009). ULK-Atg13-FIP200 complexes mediate mTOR signaling to the autophagy machinery. Mol Biol Cell.

[23] Liu Y, Yang Q, Chen S, Li Z, Fu L (2023). Targeting VPS34 in autophagy: an update on pharmacological small-molecule compounds. Eur J Med Chem.

[24] Axe EL, Walker SA, Manifava M, Chandra P, Roderick HL, Habermann A (2008). Autophagosome formation from membrane compartments enriched in phosphatidylinositol 3-phosphate and dynamically connected to the endoplasmic reticulum. J Cell Biol.

[25] Park JM, Seo M, Jung CH, Grunwald D, Stone M, Otto NM (2018). ULK1 phosphorylates Ser30 of BECN1 in association with ATG14 to stimulate autophagy induction. Autophagy.

[26] Russell RC, Tian Y, Yuan H, Park HW, Chang YY, Kim J (2013). ULK1 induces autophagy by phosphorylating Beclin-1 and activating VPS34 lipid kinase. Nat Cell Biol.

[27] Glick D, Barth S, Macleod KF (2010). Autophagy: cellular and molecular mechanisms. J Pathol.

[28] Yu L, Chen Y, Tooze SA (2018). Autophagy pathway: cellular and molecular mechanisms. Autophagy.

[29] Joshi AS, Zhang H, Prinz WA (2017). Organelle biogenesis in the endoplasmic reticulum. Nat Cell Biol.

[30] Burman C, Ktistakis NT (2010). Regulation of autophagy by phosphatidylinositol 3-phosphate. FEBS Lett.

[31] Proikas-Cezanne T, Ruckerbauer S, Stierhof YD, Berg C, Nordheim A (2007). Human WIPI-1 puncta-formation: a novel assay to assess mammalian autophagy. FEBS Lett.

[32] Polson HE, de Lartigue J, Rigden DJ, Reedijk M, Urbé S, Clague MJ (2010). Mammalian Atg18 (WIPI2) localizes to omegasome-anchored phagophores and positively regulates LC3 lipidation. Autophagy.

[33] Geng J, Klionsky DJ. The Atg8 and Atg12 ubiquitin-like conjugation systems in macroautophagy. 'Protein modifications: beyond the usual suspects' review series. EMBO Reports 2008;9(9):859–64. 10.1038/embor.2008.163 PMC2529362 18704115

[34] Klionsky DJ, Schulman BA (2014). Dynamic regulation of macroautophagy by distinctive ubiquitin-like proteins. Nat Struct Mol Biol.

[35] Dooley HC, Razi M, Polson HE, Girardin SE, Wilson MI, Tooze SA (2014). WIPI2 links LC3 conjugation with PI3P, autophagosome formation, and pathogen clearance by recruiting Atg12-5-16L1. Mol Cell.

[36] Mizushima N, Kuma A, Kobayashi Y, Yamamoto A, Matsubae M, Takao T (2003). Mouse Apg16L, a novel WD-repeat protein, targets to the autophagic isolation membrane with the Apg12-Apg5 conjugate. J Cell Sci.

[37] Mizushima N, Yamamoto A, Hatano M, Kobayashi Y, Kabeya Y, Suzuki K (2001). Dissection of autophagosome formation using Apg5-deficient mouse embryonic stem cells. J Cell Biol.

[38] Weidberg H, Shvets E, Shpilka T, Shimron F, Shinder V, Elazar Z (2010). LC3 and GATE-16/GABARAP subfamilies are both essential yet act differently in autophagosome biogenesis. EMBO J.

[39] Kabeya Y, Mizushima N, Ueno T, Yamamoto A, Kirisako T, Noda T (2000). LC3, a mammalian homologue of yeast Apg8p, is localized in autophagosome membranes after processing. EMBO J.

[40] Ichimura Y, Kirisako T, Takao T, Satomi Y, Shimonishi Y, Ishihara N (2000). A ubiquitin-like system mediates protein lipidation. Nature.

[41] Fujita N, Itoh T, Omori H, Fukuda M, Noda T, Yoshimori T (2008). The Atg16L complex specifies the site of LC3 lipidation for membrane biogenesis in autophagy. Mol Biol Cell.

[42] Weidberg H, Shpilka T, Shvets E, Abada A, Shimron F, Elazar Z (2011). LC3 and GATE-16 N termini mediate membrane fusion processes required for autophagosome biogenesis. Dev Cell.

[43] Rubinsztein DC, Shpilka T, Elazar Z (2012). Mechanisms of autophagosome biogenesis. Curr Biology.

[44] Zhen Y, Spangenberg H, Munson MJ, Brech A, Schink KO, Tan KW (2020). ESCRT-mediated phagophore sealing during mitophagy. Autophagy.

[45] Takahashi Y, He H, Tang Z, Hattori T, Liu Y, Young MM (2018). An autophagy assay reveals the ESCRT-III component CHMP2A as a regulator of phagophore closure. Nat Commun.

[46] Flower TG, Takahashi Y, Hudait A, Rose K, Tjahjono N, Pak AJ (2020). A helical assembly of human ESCRT-I scaffolds reverse-topology membrane scission. Nat Struct Mol Biol.

[47] Tsuboyama K, Koyama-Honda I, Sakamaki Y, Koike M, Morishita H, Mizushima N (2016). The ATG conjugation systems are important for degradation of the inner autophagosomal membrane. Science (New York, NY).

[48] Jahreiss L, Menzies FM, Rubinsztein DC (2008). The itinerary of autophagosomes: from peripheral formation to kiss-and-run fusion with lysosomes. Traffic (Copenhagen, Denmark).

[49] Zhao YG, Zhang H (2019). Autophagosome maturation: An epic journey from the ER to lysosomes. J Cell Biol.

[50] Itakura E, Kishi-Itakura C, Mizushima N (2012). The hairpin-type tail-anchored SNARE syntaxin 17 targets to autophagosomes for fusion with endosomes/lysosomes. Cell.

[51] Wang Z, Miao G, Xue X, Guo X, Yuan C, Wang Z (2016). The Vici syndrome protein EPG5 Is a Rab7 effector that determines the fusion specificity of autophagosomes with late endosomes/lysosomes. Mol Cell.

[52] Diao J, Liu R, Rong Y, Zhao M, Zhang J, Lai Y (2015). ATG14 promotes membrane tethering and fusion of autophagosomes to endolysosomes. Nature.

[53] Rong Y, McPhee CK, Deng S, Huang L, Chen L, Liu M (2011). Spinster is required for autophagic lysosome reformation and mTOR reactivation following starvation. Proc Natl Acad Sci USA.

[54] Rong Y, Liu M, Ma L, Du W, Zhang H, Tian Y (2012). Clathrin and phosphatidylinositol-4,5-bisphosphate regulate autophagic lysosome reformation. Nat Cell Biol.

[55] Du W, Su QP, Chen Y, Zhu Y, Jiang D, Rong Y (2016). Kinesin 1 drives autolysosome tubulation. Dev Cell.

[56] Schulze RJ, Weller SG, Schroeder B, Krueger EW, Chi S, Casey CA (2013). Lipid droplet breakdown requires dynamin 2 for vesiculation of autolysosomal tubules in hepatocytes. J Cell Biol.

[57] Gautreau AM, Fregoso FE, Simanov G, Dominguez R (2022). Nucleation, stabilization, and disassembly of branched actin networks. Trends Cell Biol.

[58] Blanchoin L, Boujemaa-Paterski R, Sykes C, Plastino J (2014). Actin dynamics, architecture, and mechanics in cell motility. Physiol Rev.

[59] Khaitlina SY (2014). Intracellular transport based on actin polymerization. Biochemistry Biokhimiia.

[60] Molinie N, Gautreau A (2018). The Arp2/3 regulatory system and its deregulation in cancer. Physiol Rev.

[61] Fäßler F, Dimchev G, Hodirnau VV, Wan W, Schur FKM (2020). Cryo-electron tomography structure of Arp2/3 complex in cells reveals new insights into the branch junction. Nat Commun.

[62] Siton-Mendelson O, Bernheim-Groswasser A (2017). Functional actin networks under construction: the cooperative action of actin nucleation and elongation factors. Trends Biochem Sci.

[63] Schaks M, Giannone G, Rottner K (2019). Actin dynamics in cell migration. Essays Biochem.

[64] Fehrenbacher K, Huckaba T, Yang HC, Boldogh I, Pon L (2003). Actin comet tails, endosomes and endosymbionts. J Exp Biol.

[65] Cameron LA, Svitkina TM, Vignjevic D, Theriot JA, Borisy GG (2001). Dendritic organization of actin comet tails. Curr Biol CB.

[66] Weinberg J, Drubin DG (2012). Clathrin-mediated endocytosis in budding yeast. Trends Cell Biol.

[67] Anitei M, Hoflack B (2011). Bridging membrane and cytoskeleton dynamics in the secretory and endocytic pathways. Nat Cell Biol.

[68] Underhill DM, Goodridge HS (2012). Information processing during phagocytosis. Nat Rev Immunol.

[69] Aplin A, Jasionowski T, Tuttle DL, Lenk SE, Dunn WA (1992). Cytoskeletal elements are required for the formation and maturation of autophagic vacuoles. J Cell Physiol.

[70] Hamasaki M, Noda T, Baba M, Ohsumi Y (2005). Starvation triggers the delivery of the endoplasmic reticulum to the vacuole via autophagy in yeast. Traffic (Copenhagen, Denmark).

[71] Reggiori F, Monastyrska I, Shintani T, Klionsky DJ (2005). The actin cytoskeleton is required for selective types of autophagy, but not nonspecific autophagy, in the yeast Saccharomyces cerevisiae. Mol Biol Cell.

[72] He C, Song H, Yorimitsu T, Monastyrska I, Yen WL, Legakis JE (2006). Recruitment of Atg9 to the preautophagosomal structure by Atg11 is essential for selective autophagy in budding yeast. J Cell Biol.

[73] Aguilera MO, Berón W, Colombo MI (2012). The actin cytoskeleton participates in the early events of autophagosome formation upon starvation induced autophagy. Autophagy.

[74] Zhuo C, Ji Y, Chen Z, Kitazato K, Xiang Y, Zhong M (2013). Proteomics analysis of autophagy-deficient Atg7-/- MEFs reveals a close relationship between F-actin and autophagy. Biochem Biophys Res Commun.

[75] Mi N, Chen Y, Wang S, Chen M, Zhao M, Yang G (2015). CapZ regulates autophagosomal membrane shaping by promoting actin assembly inside the isolation membrane. Nat Cell Biol.

[76] Yamamoto H, Kakuta S, Watanabe TM, Kitamura A, Sekito T, Kondo-Kakuta C (2012). Atg9 vesicles are an important membrane source during early steps of autophagosome formation. J Cell Biol.

[77] Hetrick B, Han MS, Helgeson LA, Nolen BJ (2013). Small molecules CK-666 and CK-869 inhibit actin-related protein 2/3 complex by blocking an activating conformational change. Chem Biol.

[78] Coutts AS, La Thangue NB (2015). Actin nucleation by WH2 domains at the autophagosome. Nat Commun.

[79] Kast DJ, Zajac AL, Holzbaur EL, Ostap EM, Dominguez R (2015). WHAMM directs the Arp2/3 complex to the ER for autophagosome biogenesis through an actin comet tail mechanism. Curr Biol.

[80] Monastyrska I, He C, Geng J, Hoppe AD, Li Z, Klionsky DJ (2008). Arp2 links autophagic machinery with the actin cytoskeleton. Mol Biol Cell.

[81] Moreau K, Ghislat G, Hochfeld W, Renna M, Zavodszky E, Runwal G (2015). Transcriptional regulation of Annexin A2 promotes starvation-induced autophagy. Nat Commun.

[82] Dai A, Yu L, Wang HW (2019). WHAMM initiates autolysosome tubulation by promoting actin polymerization on autolysosomes. Nat Commun.

[83] Coutts AS, La Thangue NB (2016). Regulation of actin nucleation and autophagosome formation. Cell Mol Life Sci.

[84] Qualmann B, Kessels MM (2009). New players in actin polymerization–WH2-domain-containing actin nucleators. Trends Cell Biol.

[85] Rotty JD, Wu C, Bear JE (2013). New insights into the regulation and cellular functions of the ARP2/3 complex. Nat Rev Mol Cell Biol.

[86] Stradal TE, Scita G (2006). Protein complexes regulating Arp2/3-mediated actin assembly. Curr Opin Cell Biol.

[87] Kirkbride KC, Sung BH, Sinha S, Weaver AM (2011). Cortactin: a multifunctional regulator of cellular invasiveness. Cell Adh Migr.

[88] Campellone KG, Welch MD (2010). A nucleator arms race: cellular control of actin assembly. Nat Rev Mol Cell Biol.

[89] Boczkowska M, Rebowski G, Petoukhov MV, Hayes DB, Svergun DI, Dominguez R. X-ray scattering study of activated Arp2/3 complex with bound actin-WCA. Structure (London, England : 1993). 2008;16(5):695–704. 10.1016/j.str.2008.02.013 PMC2849165 18462674

[90] Weaver AM, Heuser JE, Karginov AV, Lee WL, Parsons JT, Cooper JA (2002). Interaction of cortactin and N-WASp with Arp2/3 complex. Curr Biol.

[91] Helgeson LA, Nolen BJ. Mechanism of synergistic activation of Arp2/3 complex by cortactin and N-WASP. eLife. 2013;2:e00884. 10.7554/eLife.00884 PMC3762189 24015358

[92] Pant K, Chereau D, Hatch V, Dominguez R, Lehman W (2006). Cortactin binding to F-actin revealed by electron microscopy and 3D reconstruction. J Mol Biol.

[93] Weaver AM, Karginov AV, Kinley AW, Weed SA, Li Y, Parsons JT (2001). Cortactin promotes and stabilizes Arp2/3-induced actin filament network formation. Curr Biol.

[94] Lee PP, Lobato-Márquez D, Pramanik N, Sirianni A, Daza-Cajigal V, Rivers E (2017). Wiskott-Aldrich syndrome protein regulates autophagy and inflammasome activity in innate immune cells. Nat Commun.

[95] Rivers E, Rai R, Lötscher J, Hollinshead M, Markelj G, Thaventhiran J, et al. Wiskott Aldrich syndrome protein regulates non-selective autophagy and mitochondrial homeostasis in human myeloid cells. eLife. 2020;9. 10.7554/eLife.55547 PMC7673780 33135633

[96] Zavodszky E, Seaman MN, Moreau K, Jimenez-Sanchez M, Breusegem SY, Harbour ME, et al. Mutation in VPS35 associated with Parkinson’s disease impairs WASH complex association and inhibits autophagy. Nat Commun. 2014;5:3828. 10.1038/ncomms4828 PMC4024763 24819384

[97] Xia P, Wang S, Du Y, Zhao Z, Shi L, Sun L (2013). WASH inhibits autophagy through suppression of Beclin 1 ubiquitination. EMBO J.

[98] Zhang Z, Wu B, Chai W, Cao L, Wang Y, Yu Y (2016). Knockdown of WAVE1 enhances apoptosis of leukemia cells by downregulating autophagy. Int J Oncol.

[99] Hu X, Mullins RD (2019). LC3 and STRAP regulate actin filament assembly by JMY during autophagosome formation. J Cell Biol.

[100] Lee JY, Koga H, Kawaguchi Y, Tang W, Wong E, Gao YS (2010). HDAC6 controls autophagosome maturation essential for ubiquitin-selective quality-control autophagy. EMBO J.

[101] Hasegawa J, Iwamoto R, Otomo T, Nezu A, Hamasaki M, Yoshimori T (2016). Autophagosome-lysosome fusion in neurons requires INPP5E, a protein associated with Joubert syndrome. EMBO J.

[102] Rottner K, Hänisch J, Campellone KG (2010). WASH, WHAMM and JMY: regulation of Arp2/3 complex and beyond. Trends Cell Biol.

[103] Miki H, Miura K, Takenawa T (1996). N-WASP, a novel actin-depolymerizing protein, regulates the cortical cytoskeletal rearrangement in a PIP2-dependent manner downstream of tyrosine kinases. EMBO J.

[104] Antón IM, Jones GE, Wandosell F, Geha R, Ramesh N (2007). WASP-interacting protein (WIP): working in polymerisation and much more. Trends Cell Biol.

[105] Dong Y, Jin Q, Sun M, Qi D, Qu H, Wang X (2023). CLDN6 inhibits breast cancer metastasis through WIP-dependent actin cytoskeleton-mediated autophagy. J Experiment Clin Cancer Res.

[106] Gomez TS, Billadeau DD (2009). A FAM21-containing WASH complex regulates retromer-dependent sorting. Dev Cell.

[107] Duleh SN, Welch MD (2010). WASH and the Arp2/3 complex regulate endosome shape and trafficking. Cytoskeleton (Hoboken, NJ).

[108] King JS, Gueho A, Hagedorn M, Gopaldass N, Leuba F, Soldati T (2013). WASH is required for lysosomal recycling and efficient autophagic and phagocytic digestion. Mol Biol Cell.

[109] Xia P, Wang S, Huang G, Du Y, Zhu P, Li M (2014). RNF2 is recruited by WASH to ubiquitinate AMBRA1 leading to downregulation of autophagy. Cell Res.

[110] Mughees M, Bano F, Wajid S (2021). Mechanism of WASP and WAVE family proteins in the progression of prostate cancer. Protoplasma.

[111] Campellone KG, Webb NJ, Znameroski EA, Welch MD (2008). WHAMM is an Arp2/3 complex activator that binds microtubules and functions in ER to Golgi transport. Cell.

[112] Shikama N, Lee CW, France S, Delavaine L, Lyon J, Krstic-Demonacos M (1999). A novel cofactor for p300 that regulates the p53 response. Mol Cell.

[113] Zuchero JB, Coutts AS, Quinlan ME, Thangue NB, Mullins RD (2009). p53-cofactor JMY is a multifunctional actin nucleation factor. Nat Cell Biol.

[114] Schlüter K, Waschbüsch D, Anft M, Hügging D, Kind S, Hänisch J (2014). JMY is involved in anterograde vesicle trafficking from the trans-Golgi network. Eur J Cell Biol.

[115] Schnoor M, Stradal TE, Rottner K (2018). Cortactin: cell functions of a multifaceted actin-binding protein. Trends Cell Biol.

[116] Pantoom S, Pomorski A, Huth K, Hund C, Petters J, Krężel A, et al. Direct Interaction of ATP7B and LC3B Proteins Suggests a Cooperative Role of Copper Transportation and Autophagy. Cells. 2021;10(11). 10.3390/cells10113118 PMC8625360 34831341

[117] Coutts AS, Pires IM, Weston L, Buffa FM, Milani M, Li JL (2011). Hypoxia-driven cell motility reflects the interplay between JMY and HIF-1α. Oncogene.

[118] Salvi A, Thanabalu T (2017). Expression of N-WASP is regulated by HiF1α through the hypoxia response element in the N-WASP promoter. Biochem Biophys Reps.

[119] Wei J, Zhu K, Yang Z, Zhou Y, Xia Z, Ren J (2023). Hypoxia-induced autophagy is involved in radioresistance via HIF1A-Associated Beclin-1 in glioblastoma multiforme. Heliyon.

[120] Li Q, Ni Y, Zhang L, Jiang R, Xu J, Yang H (2021). HIF-1α-induced expression of m6A reader YTHDF1 drives hypoxia-induced autophagy and malignancy of hepatocellular carcinoma by promoting ATG2A and ATG14 translation. Signal Transduct Target Ther.

[121] Wang K, Kong F, Qiu Y, Chen T, Fu J, Jin X (2023). Autophagy regulation and protein kinase activity of PIK3C3 controls sertoli cell polarity through its negative regulation on SCIN (scinderin). Autophagy.

[122] Zhang Y, Cui Y, Wang L, Han J (2020). Autophagy promotes osteoclast podosome disassembly and cell motility athrough the interaction of kindlin3 with LC3. Cell Signal.

